# ABC transportome inventory of human pathogenic yeast *Candida glabrata*: Phylogenetic and expression analysis

**DOI:** 10.1371/journal.pone.0202993

**Published:** 2018-08-28

**Authors:** Sonam Kumari, Mohit Kumar, Nitesh Kumar Khandelwal, Priya Kumari, Mahendra Varma, Poonam Vishwakarma, Garima Shahi, Suman Sharma, Andrew M. Lynn, Rajendra Prasad, Naseem A. Gaur

**Affiliations:** 1 Yeast Biofuel Group, International Centre for Genetic Engineering and Biotechnology, New Delhi, India; 2 Amity Institute of Integrative Science and Health, Amity University Gurgaon, Haryana, India; 3 School of Computational and Integrative Sciences, Jawaharlal Nehru University, New Delhi, India; Vallabhbhai Patel Chest Institute, INDIA

## Abstract

ATP-binding cassette (ABC) is one of the two major superfamilies of transporters present across the evolutionary scale. ABC superfamily members came to prominence due to their ability to extrude broad spectrum of substrates and to confer multi drug resistance (MDR). Overexpression of some ABC transporters in clinical isolates of *Candida* species was attributed to the development of MDR phenotypes. Among *Candida* species, *Candida glabrata* is an emerging drug resistant species in human fungal infections. A comprehensive analysis of such proteins in *C*. *glabrata* is required to untangle their role not only in MDR but also in other biological processes. Bioinformatic analysis of proteins encoded by genome of human pathogenic yeast *C*. *glabrata* identified 25 putative ABC protein coding genes. On the basis of phylogenetic analysis, domain organization and nomenclature adopted by the Human Genome Organization (HUGO) scheme, these proteins were categorized into six subfamilies such as Pleiotropic Drug Resistance (PDR)/ABCG, Multi Drug Resistance (MDR)/ABCB, Multi Drug Resistance associated Protein (MRP)/ABCC, Adrenoleukodystrophy protein (ALDp)/ABCD, RNase L Inhibitor (RLI)/ABCE and Elongation Factor 3 (EF3)/ABCF. Among these, only 18 ABC proteins contained transmembrane domains (TMDs) and were grouped as membrane proteins, predominantly belonging to PDR, MDR, MRP, and ALDp subfamilies. A comparative phylogenetic analysis of these ABC proteins with other yeast species revealed their orthologous relationship and pointed towards their conserved functions. Quantitative real time PCR (qRT-PCR) analysis of putative membrane localized ABC protein encoding genes of *C*. *glabrata* confirmed their basal expression and showed variable transcriptional response towards antimycotic drugs. This study presents first comprehensive overview of ABC superfamily proteins of a human fungal pathogen *C*. *glabrata*, which is expected to provide an important platform for in depth analysis of their physiological relevance in cellular processes and drug resistance.

## Introduction

ABC superfamily also known as ‘Traffic ATPases’ is very diverse and well-studied family of proteins known to exist from prokaryotes to higher eukaryotes [1]. These proteins are well known for their role as high affinity nutrient importers in bacterial cells and rose to prominence as exporters in higher eukaryotes. ABC proteins are promiscuous translocator of wide range of substrates such as sugars, amino acids, ions, peptides, cholesterol, metabolites, and toxins across the biological membranes, which are powered by ATP hydrolysis [2]. These proteins are known to perform diverse functions and their ability to confer MDR brought them to eminence. Typically, a full ABC transporter consists of four distinct domains: two TMDs consisting of six transmembrane helices (TMHs) and two nucleotide binding domains (NBD), located on the cytosolic side of the membrane [3]. The NBDs, which couple energy of ATP hydrolysis to power drug export are highly conserved structure [4]. Each NBD contains three characteristic motifs: Walker A and Walker B motifs, which form the nucleotide-binding site, and an ABC signature sequence, or C motif, for which several functions have been proposed, including communication between the TMDs and NBDs during the transport cycle [5,6]. The conserved sequence motifs Walker A and Walker B are separated by approximately 120 amino acids residues and the signature sequence is placed between these two motifs [7]. TMDs on the other hand with its homo- or heteromeric repeats of unrelated TMHs provide three dimensional structures for substrate binding and help in transport process [8]. In contrast to NBDs which consist of conserved sub-domains, TMHs of two TMDs are of variable structure which imparts poly specificity to these transporters [9]. While two NBDs and two TMDs are required to become a full functional ABC transporter, half ABC transporters also known to exist with one each of NBD and TMD and presumably function as homo-dimers [10]. The eukaryotic ABC superfamily is classified into nine subfamilies (A to I), according to the HUGO nomenclature [11]. The newly added ABC subfamily ABCI (the prokaryotic type ABC protein subfamily) contains ABC proteins without NBD, and is confined to plant taxa [12]. ABCH subfamily is identified in the genomes of arthropods but is absent in most of the plants, animals and fungal genomes [13]. Subfamily ABCA is known to exist in animal, plant and various protists but is absent in yeast genome [14].

*Saccharomyces cerevisiae* and *Candida albicans* possess a battery of ABC proteins, which are reasonably well characterized for their role in MDR [7,15]. However, such information is lacking for an emerging pathogenic haploid yeast *C*. *glabrata*. Recent epidemiological data revealed that *C*. *glabrata* is the second most frequently isolated nosocomial species among fungal infections [16], and exist as a commensal of normal microbiota of oral cavity, gastrointestinal and genital tract in humans [17]. Notably, *C*. *glabrata* is phylogenetically distant from pathogenic *C*. *albicans* and closer to nonpathogenic yeast *S*. *cerevisiae* [18]. The clinical isolates of *C*. *glabrata* show high level of resistance towards commonly used antifungal such as azoles [19]. MDR, the acquisition of resistance towards various classes of antifungal drugs is a serious clinical challenge for candidiasis treatment. Recently, high attention was derived by ABC proteins of *C*. *glabrata* due to the fact that some of the efflux pumps encoding genes such as *CAGL0M01760g* (*CgCDR1*), *CAGL0F02717g* (*CgPDH1*) and *CAGL0I04862g* (*CgSNQ2*) are highly expressed in clinical isolates of *C*. *glabrata* and contribute to the development of azole resistance [20–24]. Earlier, *CAGL0M01760g* (*CgCDR1*) and *CAGL0F02717g* (*CgPDH1*) upregulation independent of *CgPDR1* mutation in fluconazole heteroresistant *C*. *glabrata* clinical isolates has been also reported [25]. Therefore, an in depth analysis of large members of ABC superfamily of *C*. *glabrata* is required to dissect their physiological relevance in MDR and other cellular processes.

This study represents the identification and phylogenetic analysis of proteins belonging to ABC superfamily of *C*. *glabrata*. Our analysis identified 25 putative ABC proteins, among them 18 members possesses at least one each of NBD and TMD and categorized as ABC transporters. The remaining 7 possess only NBDs, hence are grouped as soluble ABC proteins. Basal expression analysis of all ABC transporters revealed that all coding sequences were transcribed under normal laboratory conditions. Transient treatment with antimycotic drugs leads to the differential expression of some ABC transporters, implying their potential role in development of MDR. The phylogenetic analysis and expression profiles presented here will pave the way for future investigations involving molecular and biological significance of ABC transporters in pathogenic yeast *C*. *glabrata*.

## Materials and methods

### Identification and sequence retrieval

*C*. *glabrata* genome sequences were downloaded from the NCBI genome database (ftp.ncbi.nlm.nih.gov/genomes) with assembly no. ASM254v2. ABC proteins were identified by using the model ABC-tran (accession PF00005) of the Pfam database (https://pfam.xfam.org/) and the HMM search program from the HMMER package (http://hmmer.org/) using the default settings. Positive hits above the default threshold were further filtered by a cutoff, defined from the plot of scores and e-values (S1 Fig). Hits with domain score greater than 56.4 and e-value less than 1.2e-20 were considered true positives containing the NBD domain and extracted as potential ABC sequences for further analysis (Table 1).

**Table 1:** Predicted ABC proteins in *C*. *glabrata*.

RefSeq ID	ORF Name	Gene names*	Length#	UniProt Entry
XP_448240.1	*CAGL0K00363g*		1227	Q6FNF4
XP_444820.1	*CAGL0A01133g*		801	Q6FXW2
XP_449941.1	*CAGL0M13739g*	*ATM1*	727	Q6FIK3
XP_445704.1	*CAGL0E00385g*		608	Q6FVP0
XP_445860.1	*CAGL0E03982g*		1659	Q6FV84
XP_445319.1	*CAGL0C03289g*	*YBT1*	1648	Q6FWS5
XP_445834.1	*CAGL0E03355g*		1535	Q6FVB0
XP_449053.1	*CAGL0L06402g*	*YCF1*	1535	Q6FL41
XP_446375.1	*CAGL0G00242g*	*YOR1*	1477	Q6FTR9
XP_445428.1	*CAGL0D00352g*		861	Q6FWG6
XP_449450.1	*CAGL0M02387g*		856	Q6FJZ4
XP_446712.1	*CAGL0G08041g*		607	Q6FST2
XP_445278.2	*CAGL0C02343g*	*ARB1*	720	Q6FWW6
XP_445575.1	*CAGL0D03674g*		1186	Q6FW19
XP_448674.1	*CAGL0K10472g*		752	Q6FM70
XP_445123.1	*CAGL0B03487g*	*TEF3*	1045	O93796
XP_447598.1	*CAGL0I08019g*		1285	Q6FQ96
XP_449421.1	*CAGL0M01760g*	*CDR1*	1499	Q6FK23
XP_447461.1	*CAGL0I04862g*	*SNQ2*	1507	Q6FQN3
XP_446088.1	*CAGL0F02717g*	*PDH1/CGR1*	1542	O74208
XP_449665.1	*CAGL0M07293g*		1515	Q6FJC9
XP_446033.1	*CAGL0F01419g*	*AUS1*	1398	Q6FUR1
XP_449114.1	*CAGL0L07744g*		1055	Q6FKY0
XP_446582.1	*CAGL0G05093g*		544	Q6FT62
XP_448758.1	*CAGL0K12474g*		294	Q6FLY6

* Name of genes given in the NCBI database

# Length of proteins in amino acids

### Multiple sequence alignment and phylogenetic analysis

Amino acid sequences of *C*. *glabrata* NBDs (accession PF00005) were extracted as per their locations in the ABC proteins and *S*. *cerevisiae* NBD sequences of ABC proteins were retrieved by UniProt (www.uniprot.org/). The complete amino acid sequences of ABC proteins of *S*. *cerevisiae*, *C*. *albicans*, *Cryptococcus neoformans*, *Kleyvuromyces lactis* and *Schizosaccharomyces pombe* were taken from previously published report [14]. ITS sequences were taken from online database SILVA (https://www.arb-silva.de/). Sequences were aligned by ClustalW with default parameters and phylogenetic trees were generated by MEGA6.06 using maximum likelihood (ML) method and poisson amino acid substitution model with 1000 bootstrap replications. Sequence identities of ABC proteins among different species were analyzed by using BLASTp (https://blast.ncbi.nlm.nih.gov/Blast.cgi?PAGE=Proteins) with default parameters.

### Topology, Chromosomal location, and localization prediction of ABC proteins

The topology of *C*. *glabrata* ABC proteins was predicted by online softwares Scan Prosite (http://prosite.expasy.org/scanprosite/) and Topocons (http://topcons.cbr.su.se/). Scan Prosite was used to recognize the NBD location and Topocons was utilized for TMH identification. Size and chromosomal location of the ABC proteins were retrieved from the Candida Genome Database (http://www.candidagenome.org/) and a circular ideogram was generated by using Circos software (http://circos.ca/software/). To predict subcellular localization of ABC proteins, LocTree3 (https://rostlab.org/services/loctree2/) and WoLF PSORT (https://www.genscript.com/wolf-psort.html) were employed with input of putative ABC proteins amino acid sequences.

### Strain and Chemicals

Two *C*. *glabrata* strains reference strain CBS138/ATCC2001 and BG2, gifted by New Jersey Medical School–Rutgers, Newark, New Jersey and Lab of fungal pathogenesis, CDFD, Hyderabad, India respectively, were used in present study. Yeast cultures were maintained in YPD broth (2% peptone, 1% yeast extract and 2% glucose) and YPD Agar (YPD broth with 2% agar). Antifungal drugs (fluconazole, amphotericin B, caspofungin and ketoconazole) were of analytical grade and purchased from Sigma Aldrich, India.

### Total RNA isolation and cDNA synthesis

Total RNA was extracted by using RNeasy Mini Kit (QIAGEN, Germany) using prescribed protocol. Briefly, overnight grown cultures of *C*. *glabrata* were diluted to 0.1 OD_600_ and grown for 4 hrs. at 30°C in YPD, followed by 60 minutes treatment with desired drugs (fluconazole, 16μg/ml; amphotericin B, 1μg/ml; caspofungin, 75ng/ml and ketoconazole, 0.5μg/ml) concentration as per MIC_50_ values. Cells were washed with PBS and total RNA was extracted as per the manufacturer’s protocol. RNA samples were quantified using nanodrop 2000 spectrophotometer (Thermo Scientific, USA) and 5μg of total RNA was used for cDNA synthesis by using RevertAid H minus first strand cDNA synthesis kit (Thermo Scientific, Lithuania).

### Quantitative real-time PCR

Quantitative gene expression profile was evaluated by using DyNAmo Flash SYBR Green qPCR kit (Thermo Scientific, Lithuania) and gene-specific primers (Table 2) including *CAGL0G09383g* (*CgTDH3*) for normalization in CFX96TM real time PCR system (Bio-Rad, USA). *CgTDH3* gene was used as reference gene because its expression was constant in presence of all tested conditions (data not shown). The basal expression level of ABC transporters was measured by comparing the Ct value of the gene with *CgTDH3*. Comparative expression profiles of ABC transporters in drug-treated versus untreated conditions were analyzed by 2^-ΔΔCt^ method [26]. qRT-PCR was performed in biological duplicate with technical triplicate.

**Table 2:** Primers used in this study.

ABC Gene ORF	Forward primers (5’ - 3’)	Reverse primers (5’ - 3’)
*CAGL0G09383g*	CCACGGTAGATACGCTGGTG	CAGAACCCCATGGCAAGTTAGC
*CAGL0F02717g*	CTTTATATGAGGCAAGACC	GAAGTTCACCAGGAAATAG
*CAGL0F01419g*	GTCACCATACACTTACTTC	CAACCATCCACCATATTC
*CAGL0M01760g*	TGATGGCTGTAAGACTATG	TCCATACTTCGTGGTAATC
*CAGL0I04862g*	GATCCAGGTGACTCTTATAC	GGATTCCCTTACCTCAAATA
*CAGL0I08019g*	CTTTAATGGATCACCAGAG	CTGGTCTTAGAGTGTATTTC
*CAGL0M07293g*	CACGATAAGAAGGTTGTATG	CACTATATGGGCAGTAGTT
*CAGL0L07744g*	CGAAATTCCTGGGTATAAG	GGTGATTGAGACACATAC
*CAGL0G00242g*	TCTTACGTGCTCTTACTC	CCATTAGTAGGCCAATTATC
*CAGL0L06402g*	CCTCTTCTACTGGTGATATTG	CCCACATAGAATGACCTAAA
*CAGL0C03289g*	GTCGACTATGACAAGATTC	ATCCAACTCTCCACTATC
*CAGL0E03982g*	CTCTCACGTATGCTATTTC	AGTAGTGATAGTACCTTCG
*CAGL0E03355g*	CACAGGAAGGAAACTATG	GAGAGAGCATCTTCTAGT
*CAGL0D00352g*	GGTGTTCAGCAAAGATTAG	CAGACAGATATAACCGAGAT
*CAGL0M02387g*	CCTCCCAGCTATTCTATTC	CTAGTCTTGCGACAAATAAG
*CAGL0A01133g*	CTAAGAGTCCTAAGTGAAAG	GAACGAGATACCACATAAG
*CAGL0M13739g*	CTGTACCTCTGAACTTCT	CCATCACTCTTGCTTATAC
*CAGL0E00385g*	GGGTTACCTATCACATTC	GACCCAGTCCTATAATAAC
*CAGL0K00363g*	CCAACACTATTCAACGATAC	TTCCACCAGAACCTATTC

### Statistical analysis

The statistical analysis was performed by GraphPad Prism 6. Data obtained were expressed as means ± SDs. The significance of differences between control and experimental groups were analyzed by using two-way ANOVA and statistical significant differences relative to untreated condition (YPD) were determined by Sidak's multiple comparisons test with p value <0.0001.

## Results and discussion

### Identification of ABC proteins in *C*. *glabrata*

To investigate putative ABC proteins, the HMM profile of the ABC transporter domain (accession PF00005) was utilized as the queries to search against 5213 protein coding ORFs in *C*. *glabrata* genome. Initially, a total of 54 proteins consisting NBD domain in the range of 150±20 amino acids were found. Since, NBD is a member of the Rossman fold superfamily of nucleotide binding proteins, and to ensure that only NBD containing proteins are detected, a threshold was determined from the plot of domain scores and e-values (S1 Fig), and a large inflection at a score of 56.4 and e-value of 1.2 e-20 was observed. Further, examination of sequences below this score and with a higher e-value showed that canonical motifs associated with NBD were absent in 29, out of these 54 proteins. Hence, only 25 proteins with higher score and lower e-value than the set threshold were considered for further study (Table 1).

### Phylogenetic analysis, domain organization and subfamily prediction

**Fig 1:**
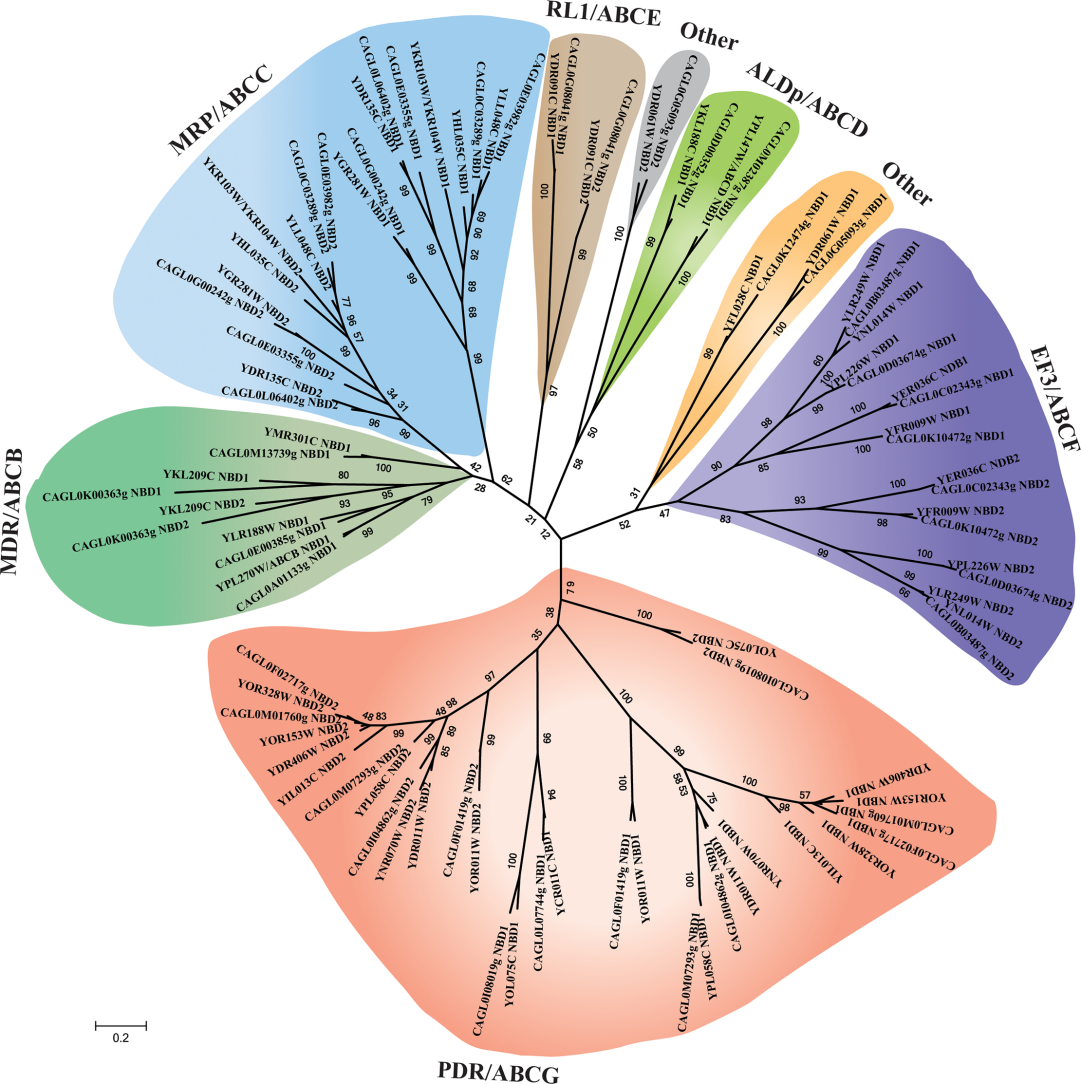
A schematic tree depicting the phylogenetic relationship among ABC subfamilies. NBDs of *C*. *glabrata* and *S*. *cerevisiae* ABC proteins were used in ML analysis. Clustering of *C*. *glabrata* NBDs is consistent with NBDs of *S*. *cerevisiae* ABC proteins. C-terminal NBDs of ABCC/MRP subfamily members clustered with ABCB/MDR subfamily NBDs. Phylogenetic tree is represented in radial form.

The complete genome sequence and phylogenetic analysis of *S*. *cerevisiae* and *C*. *albicans* identified 30 and 26 distinct ABC protein encoding genes, respectively [27]. Considering close phylogenetic similarity of *C*. *glabrata* with *S*. *cerevisiae* and to predict subfamilies, an unrooted phylogenetic tree was constructed by aligning NBDs of *C*. *glabrata* and *S*. *cerevisiae* ABC proteins as described in methods. Based on resemblance in domain organization with *S*. *cerevisiae*, putative ABC proteins of *C*. *glabrata* were segregated into six major clusters and were assigned to MDR/ABCB, MRP/ABCC, ALDp/ABCD, RLI/ABCE, EF3/ABCF and PDR/ABCG subfamilies (Fig 1). The domain organization of *C*. *glabrata* ABC proteins was consistent with that of *S*. *cerevisiae* in ML tree, wherein N- and C- terminal NBDs are segregated separately; indicating that full transporters could be the outcome of duplication of half transporters.

**Fig 2:**
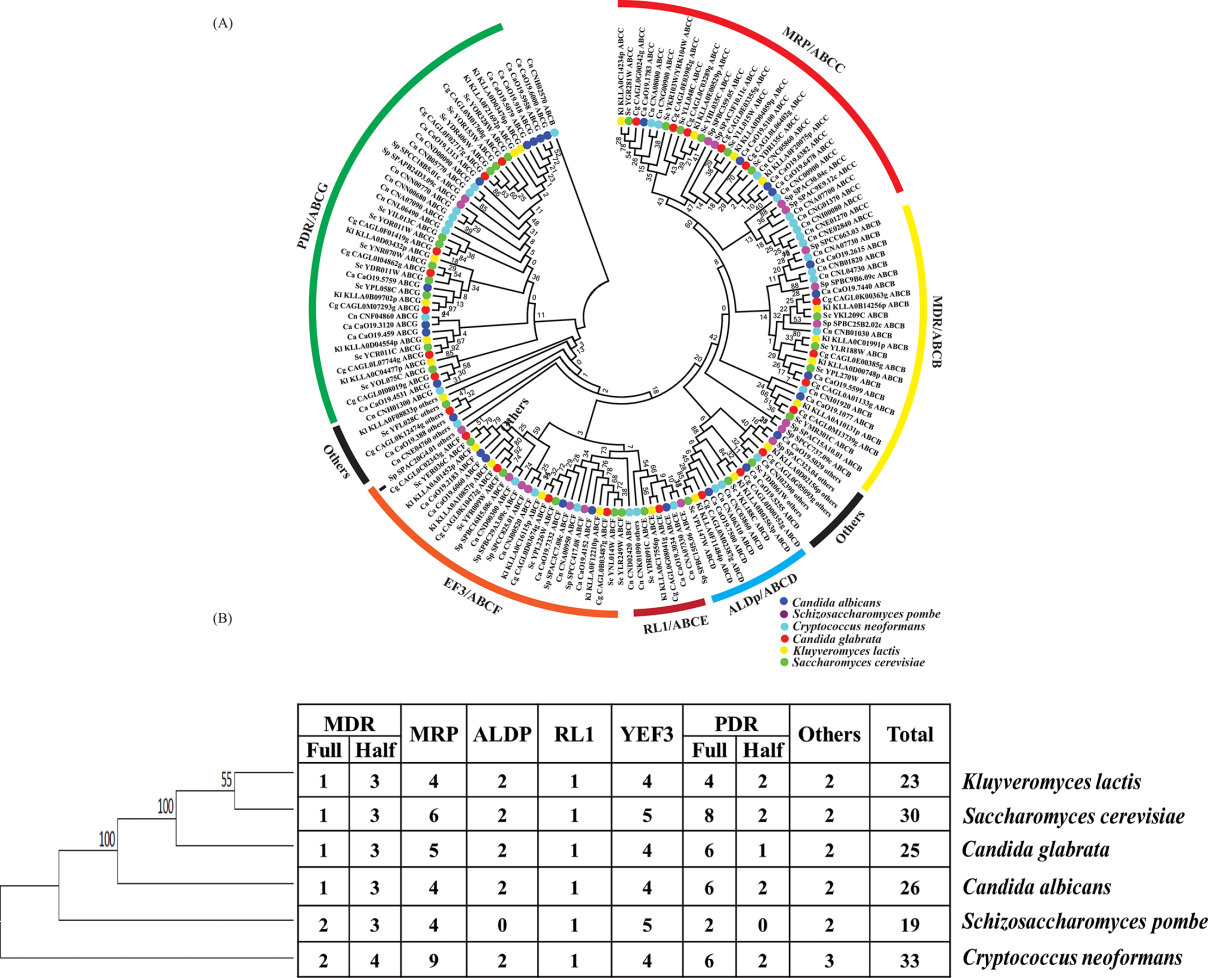
Comparison of ABC proteins in different yeast species. (**A)** Phylogenetic tree of the ABC proteins of six yeast species: The analysis was performed with putative *C*. *glabrata* ABC protein sequences with *S*. *cerevisiae*, *C*. *albicans*, *C*. *neoformans*, *K*. *lactis* and *S*. *pombe*. The numbers on the branches indicate the percentage of bootstrap support from 1000 replicates. The ABC subfamilies are identified based on known subfamilies in fungal species. (**B)** Distribution of ABC proteins among fungal species: *C*. *glabrata* harbors less number of ABC proteins as compared to *S*. *cerevisiae*, *C*. *neoformans* and *C*. *albicans* but more in number as compared to *K*. *lactis* and *S*. *pombe*. Organism phylogeny presented based on their ITS sequences.

To attain a better understanding of evolutionary relationship in ABC proteins among yeast species another phylogenetic tree was constructed by using complete ABC proteins of six representative yeast species (*S*. *cerevisiae*, *C*. *albicans*, *C*. *neoformans*, *K*. *lactis* and *S*. *pombe*) including *C*. *glabrata* (Fig 2A). Among them, *S*. *pombe* possess the least member of ABC proteins (19 ABC proteins) while *C*. *neoformans* has maximum (33 ABC proteins) ABC protein members (Fig 2B). Notably, *S*. *pombe* was distinct with regard to a number of subfamilies since it does not have any member protein belonging to ALDp/ABCD subfamily. Our analysis further revealed that phylogenetically *C*. *glabrata* ABC subfamily proteins are closer to *S*. *cerevisiae* and *K*. *lactis* ABC proteins. Interestingly, in addition to the NBD, members of each family also have conserved amino acid sequences, which could help in providing a comparative evolutionary orthologous relationship among various yeast species. Considering almost similar size of *C*. *glabrata* (12.3Mb) and *S*. *cerevisiae* (12.1Mb) genome, it is notable that the former possess lesser number of ABC proteins, which could suggest loss of some of the genes during whole genome duplication (WGD) event [28]. In *S*. *cerevisiae* and *C*. *glabrata* ABCG/PDR family forms the largest subfamily, although it has significantly lower number in *C*. *glabrata*. Among subfamilies, preferentially MDR/ABCB, ALDp/ABCD and RLI/ABCE family members were retained during duplication event, indicating their significance in cell physiology.

**Fig 3:**
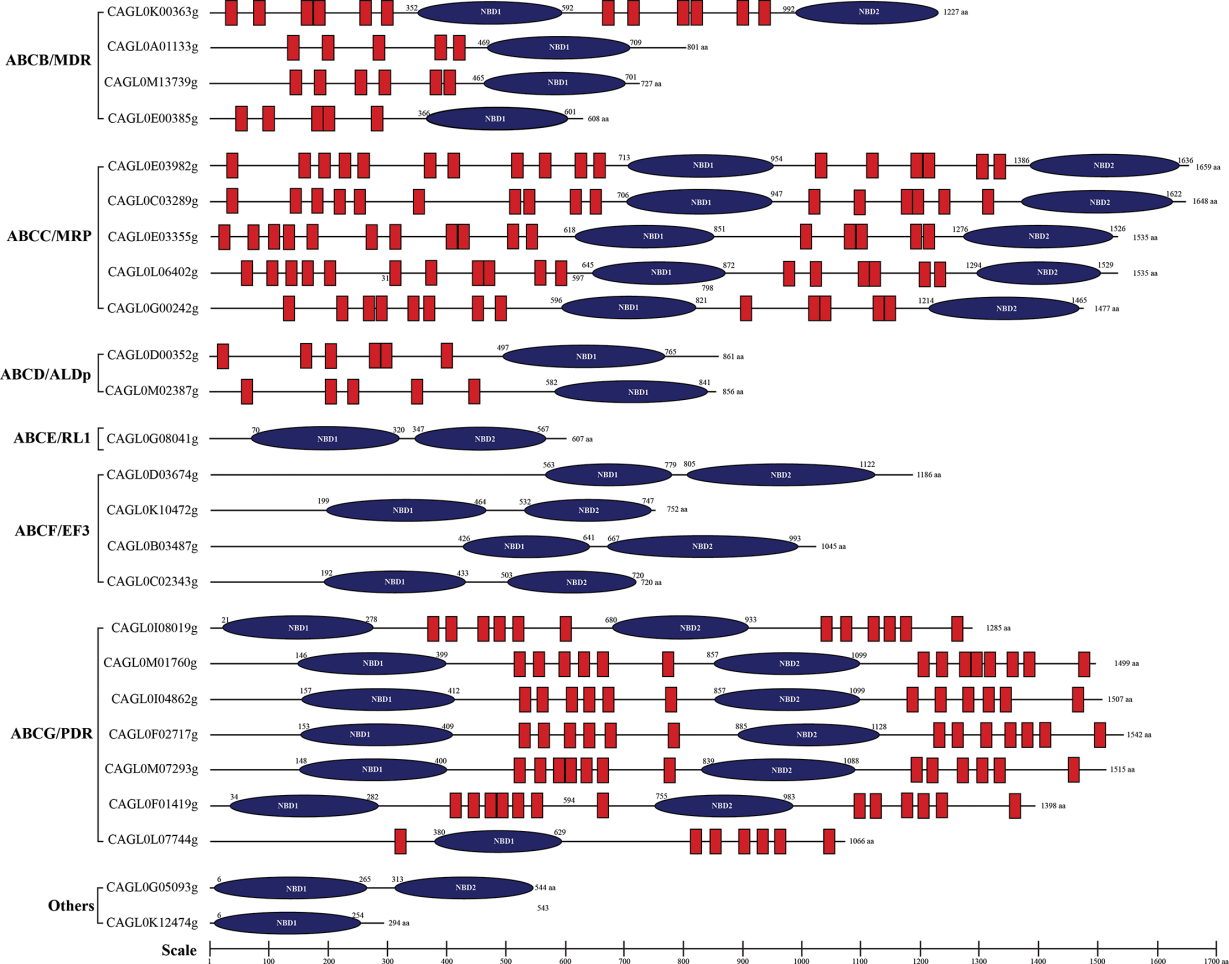
Pictorial depiction of ABC protein topology in *C*. *glabrata*. Topology prediction was performed by online softwares Scan Prosite and Topocons: All the subfamily members of ABC proteins harbor either one or two NBD. The MDR/ABCB subfamily displayed forward topology and consist of both full and half transporters, while members of the PDR/ABCG subfamily displayed reverse topology. One of the MRP/ABCC family members *CAGL0G00242g* (*CgYOR1*) does not contain extra set of transmembrane domains. ALDP/ABCD member proteins are half transporter with same topology as MDR half transporters. Members of the RLI/ABCE, EF3/ABCF and other soluble ABC proteins do not contain transmembrane domains. The scale indicates the number of amino acids.

ABC proteins have two characteristic topological structures: TMD and NBD. The presence of hydrophobic TMD in the protein makes it an eligible candidate of membrane-localized proteins, while presence of only NBD makes it a putative soluble protein. To investigate whether *C*. *glabrata* ABC proteins follow the same domain organization as reported in other organisms, the position of NBDs and TMDs were determined in each of the ABC protein sequences by using Topocons and Scan Prosite. Based on the order of NBDs and TMDs, the ABC proteins belonging to the subfamilies ABCB/MDR, ABCC/MRP, ABCD/ALDp were predicted to possess forward topology, wherein TMD precedes the NBD (TMD-NBD), while the members of ABCG/PDR subfamily have reverse orientation, where TMD follows NBD (NBD-TMD) [29] (Fig 3). A typical TMD is comprised of 6 to 10 TMHs [30], however the prediction with different topology predicting softwares indicated the presence of a variable number of TMH in ABC proteins containing TMDs (S1 File). All the proteins with only NBDs in primary structure belong to ABCE/RLI and ABCF/EF3 subfamilies and were predicted as soluble proteins.

### Multi drug resistance (MDR)/ABCB subfamily

The MDR subfamily members comprise of both full and half transporters with forward topology, (TMD-NBD)_2_ or (TMD-NBD). ABCB1 was the first characterized member of MDR subfamily for its ability to confer MDR in mammalian cells [1]. This subfamily is further divided into three subtypes: full transporters (involved in MDR), half transporters (involved in the transportation of peptides) and mitochondrial half membrane transporters [31]. Four ABC proteins of *C*. *glabrata CAGL0K00363g*, *CAGL0E00385g*, *CAGL0M13739g* (*CgATM1*), and *CAGL0A01133g* belong to MDR subfamily but are remain to be characterized (Fig 1). Among these *CAGL0K00363g*, the only member of MDR/ABCB full transporter in *C*. *glabrata*, was clustered with similar full transporters in other yeasts. It showed 42% sequence identity with *S*. *cerevisiae* haploid-specific ABC transporter *STE6*, the a-factor pheromone transporter [32]. The three half transporters *CAGL0M13739g* (*CgATM1*), *CAGL0A01133g*, and *CAGL0E00385g* showed an orthologous relationship with *S*. *cerevisiae* mitochondrial half transporters. *CAGL0M13739g* (*CgATM1*) is ortholog of an inner mitochondrial membrane-localized transporters of *S*. *cerevisiae* (*ATM1*), which translocate iron-sulfur (Fe/S) clusters into the cytosol [33] and has a well-defined mitochondrial targeting signal sequence. Biogenesis of iron-sulfur (Fe/S) clusters as a function of *ATM1* is highly conserved among various fungal species [14]. In *C*. *glabrata*, *CAGL0M13739g* (*CgATM1*) is involved in osmosensitivity and echinocandin sensitivity [34]. *CAGL0E00385g* and *CAGL0A01133g* are orthologs of peptide transporter *MDL1* and *MDL2*, respectively, of *S*. *cerevisiae* localized in the mitochondrial inner membrane [35].

#### Multi drug resistance associated proteins (MRP)/ABCC subfamily

None of the ABC proteins of this subfamily are characterized in *C*. *glabrata*. MRP subfamily members belonging to mammalian cells and other major groups of eukaryotes are known to efflux diverse array of toxic substrates including drugs and xenobiotics compounds outside the cell or sequester into the vacuole [36]. The unique feature of this family is to recognize glutathione (GSH)-, glucuronate- and sulfate- conjugated organic anions and are involved in detoxification processes [37]. It has also been observed that MRPs synergistically act with a large number of enzymes viz, GSH-S-transferase, UDP- glycosyltransferase to contribute resistance towards various substrates [38]. Notably, unlike MDR or PDR subfamilies, all the MRP members exist as full transporters. Five putative ABC proteins of *C*. *glabrata* were clustered into this group, among them *CAGL0E03982g*, *CAGL0C03289g* (*CgYBT1*), *CAGL0E03355g*, and *CAGL0L06402g* (*CgYCF1*) have an extra transmembrane region TMD_0_(TMD- NBD)_2_ and were considered as long MRPs, and *CAGL0G00242g* (*CgYOR1*) has topology of (TMD- NBD)_2,_ considered to be short MRP (Fig 3). *C*. *glabrata* MRP members showed phylogenetic similarity with *K*. *lactis* and *S*. *cerevisiae* ABC proteins. However, *CAGL0G00242g* (*CgYOR1*) have 71% sequence identity with *S*. *cerevisiae YOR1*, a well-studied plasma membrane localized transporter involved in transport of various organic anions including oligomycin and phospholipids [39]. *CAGL0G00242g* (*CgYOR1*) also exhibited 31% sequence identity with the only known channel human *CFTR/ABCC7* in the ABC transporter superfamily protein, where mutations in its encoding gene sequence were linked to cystic fibrosis [40]. *CAGL0C03289g* (Cg*YBT1*) has a sequence identity of 53% with *S*. *cerevisiae YBT1*. *CAGL0G00242g* (Cg*YOR1*) and *CAGL0C03289g* (Cg*YBT1*), both were found to be upregulated in azole resistant lab mutant as well as in azole resistant clinical isolates [41,42]. 42% sequence identity of *CAGL0L06402g* (Cg*YCF1*) was observed with human *MRP1* which is involved in endobiotics and xenobiotics extrusion [43]. Previous report also suggested the upregulation of *CAGL0L06402g* (Cg*YCF1*), *CAGL0C03289g* (Cg*YBT1*) *and CAGL0G00242g* (Cg*YOR1*) in the petite strain isolates [44].

#### Adrenoleukodystrophy protein (ALDp)/ABCD)

ALDp/ABCD subfamily proteins found in all eukaryotic organisms with an exception to some plants predominantly exist as half transporters [45]. Half ALDp members homogenously or heterogeneously dimerize to become functional [46]. In *C*. *glabrata CAGL0M02387g* and *CAGL0D00352g* belong to this subfamily which showed orthology with *S*. *cerevisiae PXA1* and *PXA2*, respectively. ALDps are peroxisomal membrane localized proteins in *S*. *cerevisiae*, involved in long and branched chain fatty acids import or their conjugate fatty acyl-CoA derivatives transport to peroxisome [47]. Interestingly, mutations in either *PXA1* or *PXA2* make cells incompetent to grow on oleic acid, suggesting that probably they function as a heterodimer [48]. Moreover, both ALDps of *C*. *glabrata* such as *CAGL0M02387g* and *CAGL0D00352g*, showed 32% and 33% sequence identity with human ABCD2 and 31% and 32% identity with *ABCD1*. The human *ABCD1* and *ABCD2* are involved in translocation of polyunsaturated VLCFA-CoA and mutation in these genes result in a human disease, X-linked Adrenoleukodystrophy (X-ALD) [49]. The clear evolutionary orthologous relationship between this family members in various species strongly point to their conserved functions.

#### RNase L Inhibitor (RLI)/ABCE subfamily

The RLI/ABCE subfamily proteins have a characteristic pair of linked NBDs with no TMDs. These soluble proteins are predicted to have a role in biological processes rather than in membrane transport [50]. In human *ABCE1*, an RLI protein is associated with polyribosomes and functions to inhibit RNase L to initiate translation [51]. *CAGL0G08041g*, the only member of this family in *C*. *glabrata*, displayed NBD-NBD topology and has a characteristic ferredoxin iron-sulfur type binding domain (4Fe-4S) with the consensus sequence of C-X-{P}-C-X(2)-C-{CP}-X(2)-C-[PEG], (pfam00037, where four C’s are the 4Fe-4S center) which is a typical motif found in nucleic acid binding proteins. *CAGL0G08041g* showed high sequence identity with *S*. *cerevisiae RLI1* (89%) and *K*. *lactis KLLA0C17556p* (88%). *RLI1* of *S*. *cerevisiae*, an iron-sulfur (4Fe-4S) protein, is involved in maturation of ribosomal subunit, translation initiation through interaction with eIF3 complex and also have a role in translational termination [52], however, this protein remains uncharacterized in *C*. *glabrata*.

#### Elongation factor 3 (EF3)/ABCF subfamilies

The members of this subfamily are topologically similar to RLI subfamily proteins with paired NBDs without TMDs. In human and yeast species, *EF3* is involved in several aspects of translation such as ribosome biogenesis, translational control of gene expression, exporting mRNA into cytoplasm, or act as a translational elongation factor [14]. A total of four members namely, *CAGL0K10472g*, *CAGL0C02343g*, *CAGL0D03674g*, and *CAGL0B03487g* (*CgTEF3*) of ABC proteins in *C*. *glabrata* belonged to this subfamily. *CAGL0B03487g* (*CgTEF3*) and *CAGL0C02343g* showed 88% and 90% sequence identity with *S*. *cerevisiae TEF3* and *ARB1*, respectively. In *C*. *glabrata*, *CAGL0B03487g* (*CgTEF3*) is an essential gene for growth and at protein level, it has been reported to be down regulated at alkaline pH [53,54]. The best characterized member of this subfamily in *S*. *cerevisiae* is *GCN20*, which mediates the activation of eIF-2α kinase [55] and showed 89% sequence identity with *CAGL0K10472g*. All members of this family are remain uncharacterized in *C*. *glabrata*.

#### Pleiotropic Drug Resistance (PDR)/ABCG subfamily

PDR/ABCG subfamily is ubiquitous throughout the plants, fungi and animal kingdom but absent in bacteria. In animals, PDR subfamily members exist as half transporters and are homologous to the white-brown-complex (WBC) family transporter of *Drosophila*. In previous report, it is suggested that there could be two possibilities of generation of PDR/ABCG subfamily, 1) by fusion of independent NBD and TMD, because it is the only subfamily in which NBD precedes the TMD (reverse topology) or 2) by direct origin from the central portion of a member of the ABCA, ABCB, or ABCC subfamilies [56]. Notably, PDR/ABCG subfamily members play important roles in extruding a variety of xenobiotics including agricultural fungicides, azoles, mycotoxins, herbicides, and antifungal drugs [57]. Similar to *S*. *cerevisiae* and *C*. *albicans*, PDR/ABCG subfamily with 7 members; *CAGL0F02717g* (*CgPDH1*), *CAGL0F01419g* (*CgAUS1*), *CAGL0M01760g* (*CgCDR1*), *CAGL0I04862g* (*CgSNQ2*), *CAGL0I08019g*, *CAGL0M07293g*, and *CAGL0L07744g* represents the largest group in *C*. *glabrata* wherein, all the members except *CAGL0L07744g* are full transporters. Importantly, *CAGL0M01760g* (*CgCDR1*), *CAGL0I04862g* (*CgSNQ2*) and *CAGL0F02717g* (*CgPDH1*) have been shown to play major role in the development of MDR phenotypes in clinical isolates and petite mutants of *C*. *glabrata* [58–61]. These three transporters are regulated by transcription factor PDR1 and their upregulation is linked to the azole resistance [24]. *CAGL0F01419g* (*CgAUS1*) is a sterol transporter and protects cells against azole antifungals in the presence of serum [62,63]. These *C*. *glabrata* genes showed sequence similarity with human ABCG members involved in exporting anti-cancerous drugs and cholesterol transport. Other members of PDR subfamily are poorly characterized in *C*. *glabrata*. However, their functions can be predicted based on sequence identity with PDR/ABCG members of other yeast species. For instance, *CAGL0M07293g* is an ortholog of *S*. *cerevisiae PDR12* with 85% sequence identity, which is involved in organic acid transport [64], while *CAGL0I08019g* with 36% sequence identity to *CDR6/ROA1* of *C*. *albicans* governs resistance to azoles [65]. The half transporter *CAGL0L07744g* shows 67% sequence identity with *S*. *cerevisiae ADP1*, the first identified half transporter belongs to the WBC family of *Drosophila* [66].

#### Others

Soluble ABC proteins that do not cluster into any specified subfamily has been kept under the category of others. In *C*. *glabrata* two proteins, *CAGL0G05093g* and *CAGL0K12474g* come into this category with two NBD and one NBD, respectively. *CAGL0G05093g* showed an orthologous relationship with uncharacterized protein in *S*. *cerevisiae* (*YDR061W*) and have 32% sequence identity with *C*. *albicans MODF*. *CAGL0K12474g* gene has orthologous relationship with *CAF16* of *S*. *cerevisiae* which is known to be a part of CCR4-NOT regulatory complex involved in transcriptional control of gene regulation [67].

### Chromosomal position and localization prediction

**Fig 4:**
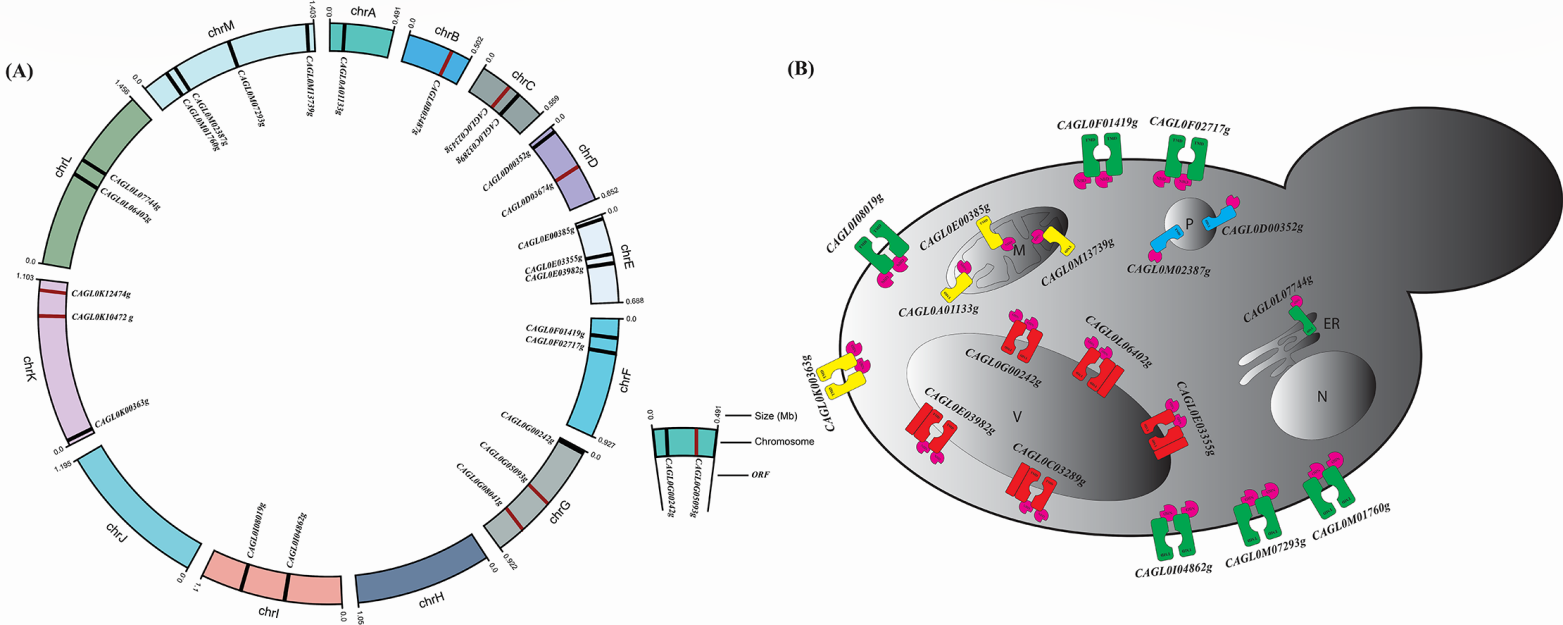
Chromosomal location and subcellular localization of ABC proteins in *C*. *glabrata*. **(A)** *C*. *glabrata* chromosomes (chrA—chrM) are displayed in circular ideogram. Black color indicates location of ABC transporter and dark maroon indicates soluble ABC proteins location. Chromosome M harbors maximum number of ABC genes and Chromosome H and J do not contain ABC genes. (**B**) Subcellular localization of the ABC transporters were predicted by LocTree3 and WoLF PSORT: Only membrane proteins are depicted.

*C*. *glabrata* with haploid genome consists of 13 chromosomes (Chr A to Chr M) [18]. Chr M harbors maximum number of ABC protein encoding genes (4 genes) followed by Chr E, Chr G and Chr K, each possess 3 genes. Each of Chr C, Chr D, Chr F, Chr I and Chr L accommodate 2 genes. Each Chr A and Chr B reside only 1 gene, however, Chr H and Chr J do not contain any ABC protein coding gene (Fig 4A).

Subcellular localization (SCL) of a protein is important to elucidate the potential function in various cellular processes. It is anticipated that proteins localized in the same cellular compartment of different organisms could perform similar type of function. SCLs of ABC proteins of *C*. *glabrata* were analysed by using online software LocTree3. The results obtained suggested that most of the *C*. *glabrata* MDR/ABCB members are localized in mitochondrial membrane and MRP/ABCC members are localized in vacuolar membrane (VM). As expected, ALDp/ABCD members were predicted to be localized on peroxisomal membrane. ABC proteins of PDR/ABCG subfamily were predicted in plasma membrane (PM) except *CAGL0L07744g*, which was found to be localized on endoplasmic reticulum (ER) (Fig 4B). Previous reports supported our localization prediction as two of the known PDR/ABCG members, *CAGL0M01760g* (*CgCDR1*) and *CAGL0F02717g* (*CgPDH1*) were shown to be plasma membrane localized [68]. Notably, localization prediction varies by the use of different softwares. ABC proteins, *CAGL0I08019g* and *CAGL0K00363g* belonging to PDR/ABCG and MDR/ABCB subfamilies, respectively, were predicted to be localized at the nucleus by LocTree3 software, however, WoLF PSORT predicted these proteins localization at PM. WoLF PSORT predicted *CAGL0G00242g* (*CgYOR1*) and *CAGL0L07744g* localization to PM, while LocTree3 predicted in VM and ER membranes, respectively. The predicted localization of 18 ABC membrane proteins are depicted in Fig 4B, while soluble ABC proteins are predicted to be confined to the cytosol or nucleus are listed in S2 File.

### Membrane localized ABC subfamily genes displayed variable response to drug exposure

Our long term goal is to unravel the role of membrane localized efflux pumps in development of MDR. Hence, for expression analysis, we selected 18 potential membrane localized members of the ABC superfamily. It is expected that expression level of genes may vary among strains with different genetic background and source of isolation. Therefore, in this study we evaluated the expression level of ABC transporter genes between the two strains of *C*. *glabrata*: a reference strain CBS138/ATCC2001 and a widely used clinical isolate BG2.

**Fig 5:**
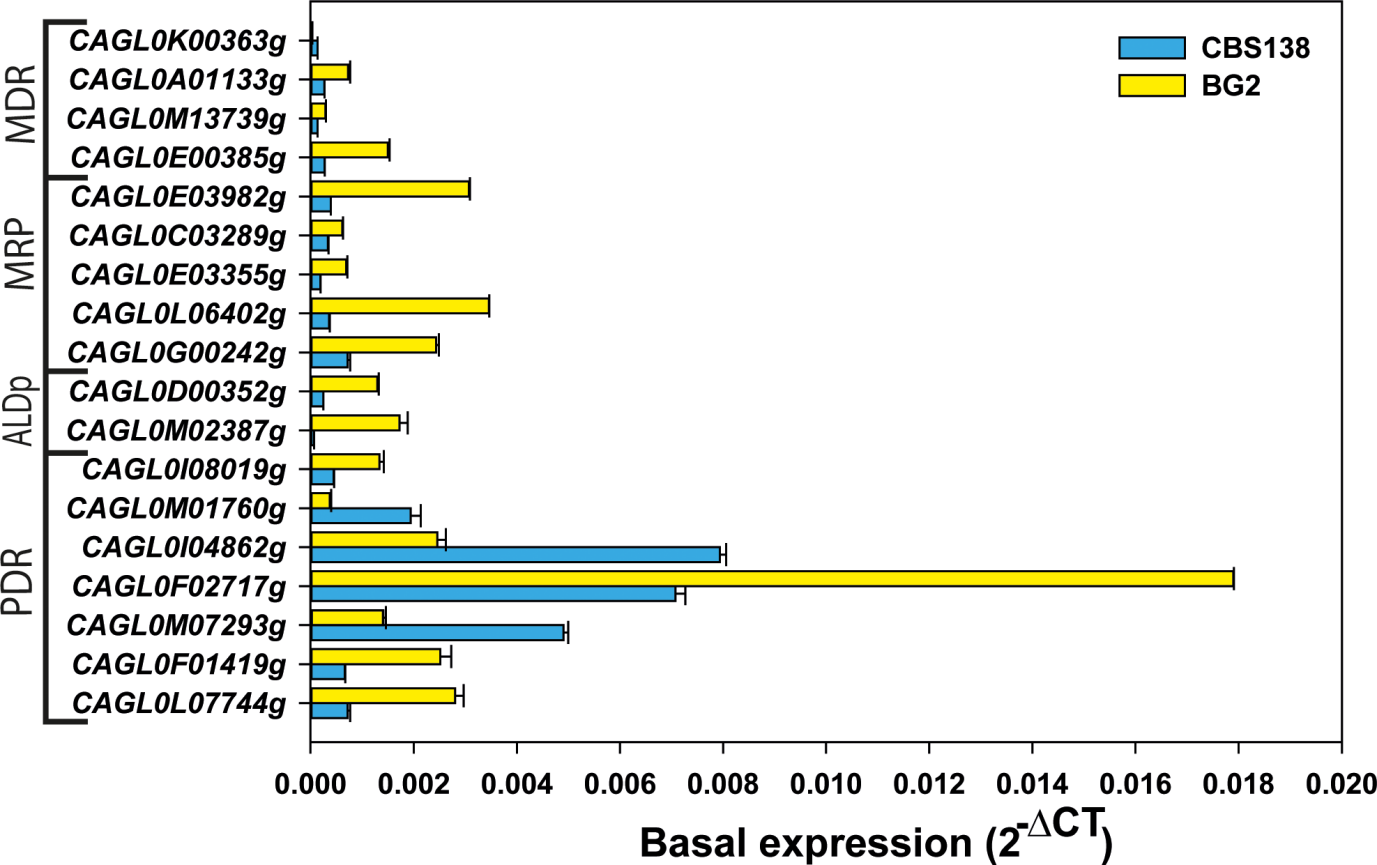
Comparative basal expression of ABC transporters in CBS138/ATCC2001 and BG2 strains. The basal expression of ABC transporters were tested by qRT-PCR in log phase grown cell under laboratory conditions (YPD, 30°C) and data was measured in 2^-ΔCT^ by normalizing with housekeeping gene *CgTDH3*. Among all ABC transporters *CAGL0K00363g* exhibited minimum expression level in both the strains.

**Fig 6:**
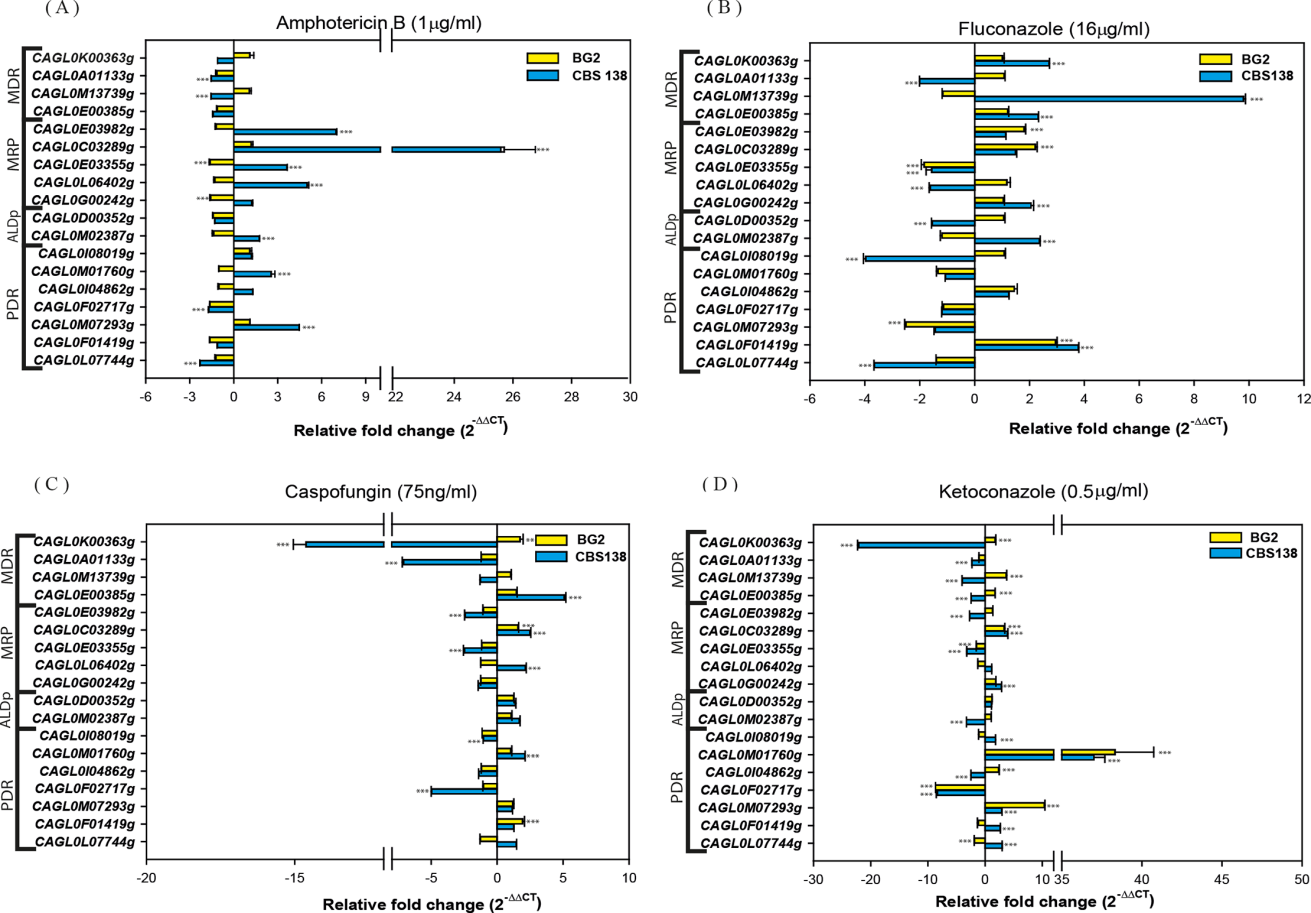
Comparative expression of ABC transporters encoding genes after transient drug exposure of CBS138/ATCC2001 and BG2 strains. The expression level was tested by qRT-PCR and data was measured by 2^-ΔΔCT^ method in the presence of amphotericin B (A), fluconazole (B), caspofungin (C), and ketoconazole (D). Transporters behaved differently in these four conditions. Expression data were assessed by two-way ANOVA and statistical significant differences relative to untreated condition (YPD) were determined by Sidak's multiple comparisons test and are indicated as stars with p value <0.0001.

Initially, we tested the basal expression level of these gene under laboratory growth conditions. qRT-PCR analysis of the mid-log phage grown cells in YPD media at 30°C confirmed that all selected ABC transporter were transcribed under normal growth condition in both the strains. However, the degree of basal expression showed considerable variation in these strains. Since strain BG2 was an isolate from a patient with initial exposure to fluconazole treatment, expectedly, in comparison to CBS138/ATCC2001, its encoding transporter genes were upregulated at basal level (Fig 5). Therefore, after transient drug exposure, the ABC transporter genes of BG2 show lesser changes in their expression. But in CBS138/ATCC2001 strain, the transient drug induction yielded stronger response and comparatively showed higher changes in expression pattern of ABC genes in all tested condition (Fig 6A–6D).

Frequently, enhanced expression of several ABC superfamily genes was directly linked to the acquisition of drug resistance among different yeast species. Earlier, several reports demonstrated that in clinical MDR isolates of *C*. *glabrata*, ABC transporters such as *CAGL0M01760g* (*CgCDR1*), *CAGL0I04862g* (*CgSNQ2*) and *CAGL0F02717g* (*CgPDH1*) were highly expressed and their expression varied in laboratory strains during transient drug induction and environmental conditions [69,70]. For evaluating the role of ABC membrane proteins coding genes, we further tested expression of all selected ABC transporters followed by 60 min exposure to azoles (fluconazole, ketoconazole), echinocandin (caspofungin) and polyene (amphotericin B) drugs. The data obtained by qRT-PCR provided an interesting insight on to the expression pattern of ABC transporters under stress conditions. For instance, all the tested drugs exhibited differential expression pattern of membrane ABC superfamily genes, implying their role in the development of drug resistance phenotypes.

Among MDR members, basal expression of *CAGL0K00363g* was lowest, in both the strains. Notably, *CAGL0K00363g* ortholog in *S*. *cerevisiae* (*STE6*) is a dedicated pheromone transporter and its human homolog (*ABCB1*) mediated MDR in various chemotherapy resistant tumors by effluxing toxic compounds [71]. Although *CAGL0K00363g* was 1.7 fold upregulated in presence of both caspofungin and ketoconazole in BG2 strain, but in CBS138/ATCC2001 strain it was down regulated to 14.6 and 22.1 fold in presence these drugs. Most of the MDR genes in CBS138/ATCC2001 strain displayed down regulation following amphotericin B, caspofungin, and ketoconazole treatment but were up regulated in fluconazole exposed cells. Interestingly, in BG2 strain the expression level of other MDR members remained unchanged during fluconazole, amphotericin B and caspofungin inductions except *CAGL0E00385g* and *CAGL0M13739g* (*CgATM1*), wherein expression level was significantly higher followed by ketoconazole exposure (Fig 6D).

Among MRP subfamily members, basal expression level of *CAGL0E03355g* was low in both the strains. *CAGL0G00242g* (*CgYOR1*) was upregulated by azoles in CBS138//ATCC2001, while, in BG2 strain the expression level was down regulated by 1.5 fold following amphotericin B treatment. However, in case of ketoconazole exposed cells, it was 1.8 fold upregulated. *CAGL0G00242g* (*CgYOR1*) ortholog in *S*. *cerevisiae* (*YOR1*) has a well demonstrated role in pleiotropic drug resistance [72]. *CAGL0C03289g* (*CgYBT1*) displayed higher expression following drug treatment in both strains. While, *CAGL0E03982g* transcript level was significantly upregulated only in fluconazole treated BG2 cells, whereas in CBS138/ATCC2001 strain, it was upregulated by amphotericin B treatment only. *CAGL0E03355g* transcript level was significantly down regulated in both tested strains followed by most of the inducing conditions. Notably, human MRP family member *ABCC1* is involved in multidrug resistance, especially during cancer and leukemia chemotherapy treatments [73] However, its ortholog *CAGL0L06402g* (*CgYCF1*) in *C*. *glabrata* does not respond to tested drug in BG2 strain but were significantly upregulated in case of amphotericin B and caspofungin exposed CBS138/ATCC2001 strain. Together, qRT-PCR analysis of MRP subfamily genes in *C*. *glabrata* similar to other yeast species do point to their involvement in drug detoxification.

Both ALDp members exhibited significantly higher expression at basal level in BG2 than in CBS138/ATCC2001. The expression level of ALDp members remained unresponsive to drug treatment in BG2. However, in CBS138/ATCC2001 strain *CAGL0M02387g* was significantly down regulated in ketoconazole and upregulated with other tested inducing conditions while *CAGL0D00352g* showed significant down regulation in fluconazole treatment.

Together, PDR subfamily member genes revealed constitutive expression, and they are the best responders to transient drug treatments. *CAGL0M01760g* (*CgCDR1*) and *CAGL0F02717g* (*CgPDH1*) have similar behavior with ketoconazole treatment in both the strains. The expression of *CAGL0M07293g*, an ortholog of *S*. *cerevisiae PDR12* (weak acid transporter) was higher following ketoconazole treatment in both the strains. The low level of expression of *CAGL0M07293g* in fluconazole resistant isolates of *C*. *glabrata* was earlier recorded by Vermitsky *et*. *al*. [41], which could be consistent with the low expression in BG2 strain. In BG2 strain *CAGL0M07293g* gene behaved differently in different azoles, where an imidazole and a triazole yielded opposite response. *CAGL0F02717g* (*CgPDH1*) was down regulated in most of the treatment condition in both the strain. Based on homology with *CaCDR1* and *CaCDR2*, it is reasonable to expect that *CAGL0F02717g* (*CgPDH1*) could be involved in phospholipid translocation and membrane lipid asymmetry maintenance [74]. As expected, fluconazole treatment induced *CAGL0F01419g* (*CgAUS1*) expression in both the strains. PDR members displayed varying degree of changes in expression, which strongly indicate their role in multi drug resistance.

## Conclusion

The study presents a first comprehensive transportome analysis of ABC proteins in *C*. *glabrata*, which led to the identification of 25 ABC proteins, representing 0.479% of protein coding genes, distributed on 11 out of 13 chromosomes. The phylogenetic comparison with other fungal species clustered these ABC members into six major subfamilies: MDR/ABCB, MRP/ABCC, ALD/ABCD, RLI/ABCE, EF3/ABCF and PDR/ABCG. The constitutive expression of all the genes encoding putative membrane localized ABC members not only confirmed their genomic presence but also reflected their biological relevance. The exposure to drugs presented variable transcriptional response among ABC membrane proteins, nonetheless, it provided sufficient clue for their potential contribution in emerging clinical drug resistance in *C*. *glabrata* isolates. Of note, majority of members of subfamilies remain unexplored. The sequence identity of *C*. *glabrata* ABC proteins with other organism could provide a basis for functional characterization of these unexplored important proteins; however, our analysis could only predict their localization and putative function. Based on the information available in *S*. *cerevisiae* and other yeasts, most of the ABC transporter genes are non-essential and hence construction of homozygous knockouts (KOs) should be realtively easy. The analysis of KOs of each member is a way forward in dissecting their role in MDR, pathogenicity and virulence. The studies so far do suggest that some of the ABC members, particularly those belonging to PDR family (*CgCDR1*, *CgPDH1* and *CgSNQ2*) have demonstrable role in developing drug resistance but considering the vast information about the role of ABC members in *C*. *albicans* and in other organisms, it is a long way before their physiological relevance of these ABC proteins could be unraveled.

## Supporting information

S1 Fig Identification of ABC proteins in *C*. *glabrata*. ABC proteins were identified by using the model ABC-tran (accession PF00005) of the Pfam database and the HMM search program. A total of 25 ABC proteins are extracted as potential ABC protein sequence. Hits with domain score greater than 56.4 and e-value less than 1.2e-20 were considered true positives containing the NBD domain.
(TIF)

S1 File Number of transmembrane helix in *C*. *glabrata* ABC protein by using different softwares. (XLSX)

S2 File Localization prediction of all *C*. *glabrata* ABC proteins. (XLSX)

S3 File NBD sequences of *C*. *glabrata* and *S*. *cerevisiae* ABC proteins. (XLSX)

S4 File Complete sequences of ABC proteins and their ITS sequences of six yeast species. (XLSX)

S5 File Chromosomal location of putative ABC protein coding genes in *C*. *glabrata*. (XLSX)

S6 File Percent identity of putative ABC proteins of *C*. *glabrata* with other species. (XLSX)
