# Supplementary figures and images for: ABC transportome inventory of human pathogenic yeast Candida glabrata: Phylogenetic and expression analysis
Source: PLoS One. 2018 Aug 28;13(8):e0202993. doi: 10.1371/journal.pone.0202993 (PMC6112666; doi:10.1371/journal.pone.0202993)

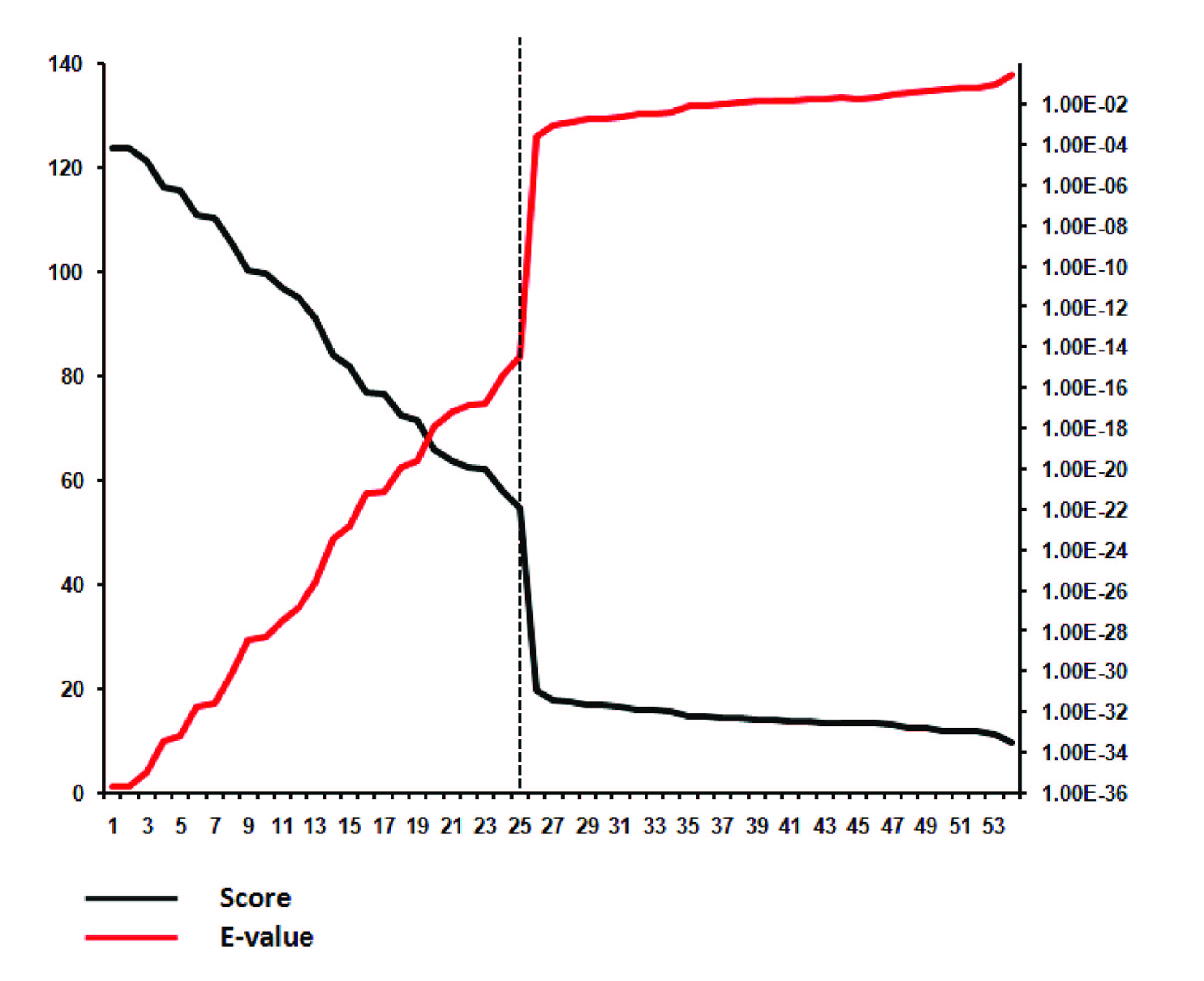

Supplement: S1 Fig — ABC proteins were identified by using the model ABC-tran (accession PF00005) of the Pfam database and the HMM search program. A total of 25 ABC proteins are extracted as potential ABC protein sequence. Hits with domain score greater than 56.4 and e-value less than 1.2e-20 were considered true positives containing the NBD domain. (TIF) [file pone.0202993.s001.tif]
